# Modified *Ziziphus spina-christi* stones as green route for the removal of heavy metals

**DOI:** 10.1038/s41598-020-76810-y

**Published:** 2020-11-25

**Authors:** Eman A. Assirey, Shadia M. Sirry, Hayfaa A. Burkani, Medhat A. Ibrahim

**Affiliations:** 1 Chemistry Department, Taibah University, Madinah, 30002 Saudi Arabia; 2 Molecular Spectroscopy and Modeling Unit, Spectroscopy Department, National Research Centre, El-Bohouth Str., 12622 Dokki, Giza, Egypt

**Keywords:** Ecology, Chemistry

## Abstract

Green routes for remediation of heavy metals are worldwide challenges to overcome pollution problems on one hand and control the adverse impact of chemicals on the other hand. Biosorption is one of the most effective methods for removing lower level of heavy metals. The idea to apply natural resources as a green method for removal of heavy metals, this route has no adverse impacts on the environment. This study investigated the ability of chemically modified *Ziziphus spina-christi* stones (ZSCs) as agriculture by-products to perform the biosorption of Pb(II), Zn(II) and Cd(II) ions from wastewater in a single and ternary metal system. The characteristic functional groups of chemically modified ZSCs were analyzed by Fourier transform infrared. In comparison with acidic ZSCs, alkali-modified ZSCs by KOH was more effective and enhanced the removal efficiency of ZSCs. Using Langmuir isotherm, the maximum adsorption capacity on the modified ZSCs for Pb(II) was 9.06 mg/g, for Zn(II) obtained by using ZSC–citric acid was 4.19 mg/g and 5.38 mg/g for Cd(II) as obtained by using ZSC–H_2_O_2_. The molecular electrostatic potential, which was calculated at B3LYP/6-31G(d,p), indicated that each metal is di-hydrated, forming a complex with two units of amino acids. This mechanism demonstrated the uptake process by ZSCs.

## Introduction

Different man-made activities with continuous effluents are considered among the main source for the contamination of aquatic ecosystems^1,2^. One of the most common constituents of these effluents the heavy metals (HMs). HMs are of growing concern, according to their accumulative and toxicity for aquatic system^3,4^. Many researchers applying different routes to control the level of HMs in the environment. Recently, a special interest has been devoted to the use of natural sorbents as an alternative to the traditional ones^5^. Biological substances have an affinity for organic and inorganic pollutants, meaning that there is enormous potential for biosorption within countless kinds of biomaterials. The high affinity of biosorbents towards metal ions is owed to their surface chemistry, which consists of many reactive functional groups characterized by their unique hydrogen bonding, which is the effective tool for remediation heavy metals from wastewater^6,7^. These functional groups are capable of binding heavy metal ions by the substitution of a hydrogen bonding with the existing metal in solution, or by the formation of metal ion-complexes through the hydrogen bond of the functional group with the given metal ions^6–9^. *Ziziphus spina-christi* refers to trees in the Rhamnaceae family; generally, in Arabic countries, the tree is called sidr and its fruit is known as nabag^10^. Its fruits, leaves, bark and roots in are used in medicine owing to their many benefits^11^. ZSCs contain protein, crude fat, fibers, carbohydrate, sugar, macro elements (potassium, calcium, sodium and magnesium), and trace elements such as copper and manganese, making the stones nontoxic^12–14^. Utilizing raw plant wastes as biosorbents can cause a low adsorption capacity^15^; however, biosorbent performance can be enhanced with several types of physical and chemical modifications^16^. The main reasons for the modification of raw biosorbents are to improve the biosorbent's resistance toward chemical and microbial degradation, biosorption selectivity, the removal of impurities, and the increase of the metal ion biosorption capacity^17,18^. Physical modification includes the appropriate sizing of the biosorbents by chopping and grinding, heat treatment (steaming and drying), ultrasonic treatment, stirring, freeze drying (lyophilization), and autoclaving^19^. Physical modification is inexpensive and simple, but it is less efficient than chemical modification^17,18^. Great improvements in the biosorption capacity can be achieved by the chemical modification of functional groups on the biosorbent surface^20^. Chemical modifications include the treatment of the biosorbents via chemical agents for washing, increasing the binding groups, the elimination of inhibiting groups and by covering the biosorbent with ionic polymers^21^. Several types of modifying agents can be used for the modification processes such as acid solutions (sulfuric acid)^22^, hydrochloric acid^23^, nitric acid^24^, base solutions (sodium hydroxide, sodium carbonate)^25^, organic compounds (formaldehyde)^26^, and oxidizing agents (hydrogen peroxide)^27^. Increasing the binding groups involves the substitution of specific functional groups on the biosorbent's surface with desirable functional groups by various chemical modification methods, such as the amination of giving functional groups, carboxylation of hydroxyl and amine groups, phosphorylation of hydroxyl groups, saponification of ester groups, halogenation and oxidation. Raw biosorbents comprise several functional groups, and only certain groups are responsible for targeting metal ions, while the other groups could interfere with the metal ions. Hence, the elimination of inhibiting groups is important for enhancing biosorption, and usually involves decarboxylation and deamination^18,19,21^.

Molecular modeling has recently shown potential application as promising tool describing the molecular interactions in many systems^28–30^. Especially those dealing with environmental interactions^31,32^. This paves the way toward, applying molecular modeling to investigating the mechanism of interaction between heavy metals and modified ZSCs.

Therefore, the present work is conducted in order to enhance the capacity of a green route based on natural material (ZSCs) which modified by different chemical modifying agents for the biosorption of Pb(II), Zn(II) and Cd(II) ions from aqueous solution in single and ternary metal systems. Isotherm models are used for the determination of their maximum sorption capacities. The active sites of interaction are indicated in terms of the electrostatic potential (ESP) and studied with the B3LYP/6-31G(d,p) molecular modeling approach.

## Materials and method

### Materials and chemicals

ZSCs were collected from Al-Madinah Al-Munawwarah fruit market. The metal salts including cadmium (II) nitrate (Cd(NO_3_)_2_.4H_2_O), lead (II) nitrate (Pb(NO_3_)_2_) and zinc nitrate Zn(NO_3_)_2_.6H_2_O used for the preparation of a stock solution of metals were purchased from Sigma-Aldrich [St Louis, MO, USA]. Sodium nitrate (NaNO_3_) (Sigma-Aldrich) was used for the adjustment of the ionic strength. Sodium acetate trihydrate (C_2_H_3_NaO_2_.3H_2_O) and acetic acid from Sigma-Aldrich were used for buffer preparation. Potassium hydroxide (KOH), hydrochloric acid (HCL), sulfuric acid (H_2_SO_4_) (Sigma-Aldrich), anhydrous citric acid (C_6_H_8_O_7_) (FlukaBioChemika) and hydrogen peroxide (H_2_O_2_) (Sigma-Aldrich) were used as modifying agents for the biosorbent.

### Preparation of modified ZSCs

ZSCs particles of size 250 μm were prepared following the previous procedure which was reported earlier^33^. They were chemically modified by treatment with 0.5 M hydrochloric acid, 0.5 M citric acid, 0.5 M sulfuric acid, 0.5 M potassium hydroxide, and 1 M hydrogen peroxide. 10 g of stone particles was shaken with 250 mL of the modifying solution at 300 rpm for two hours. ZSCs were then filtered and washed several times with distilled water using a decantation process until the pH of the solution was equal to the pH of the distilled water. ZSCs were then left to dry in the air, then finally stored in a glass bottle in the desiccator.

### Batch experiment

The experimental conditions which applied in this work, including some factors, such as the initial metal ion concentration, pH, contact time, and dose of the sorbent, were studied by batch experiment. All experiments were carried out using a series of 25 mL solutions containing an appropriate amount of each desired metal ion, buffer solutions, 0.1 M sodium nitrate solution and an adequate amount of 250 μm pore size ZSCs. These solutions were shaken using a digital orbital shaker at 300 rpm at different time intervals. The pH was adjusted using acetate buffer. The solution was filtered, and the metal ion concentration was determined according to the American Public Health Association (APHA) standard methods, 2017^34^, using inductively coupled plasma optical emission spectroscopy (ICP-OES). Each recorded reading is a sum of five replicates. The amount of adsorbed metal ions per gram of ZSCs at equilibrium *q*_*e*_(mg/g) and the removal percentage (% removal) was calculated using Eqs. (3) and (4):

1 $$q_{e}  = \frac{{\left( {C_{0}  - C_{{\text{e}}} } \right) \cdot V}}{m}$$

2 $$ {\% \hbox{Removal}} = \frac{{C_{0} - C_{e} }}{{C_{0} }} \times { }100 $$

where *C*_0_ and *C*_*e*_ are the initial and equilibrium metal ion concentration (mg L^−1^), respectively, *V* is the volume of metal ion solution (L) and m is the weight of adsorbent (g).

### Fourier transform infrared (FTIR) ANALYSIS

FTIR spectra were acquired in transmission mode using Bruker, Vertex 70, FTIR spectrometer. Spectra were recorded in the spectral region of 4000–400 cm^−1^ with 4 cm^−1^ spectral resolution and 000 co-added scans.

### Langmuir isotherm

The Langmuir isotherm is the best model for describing the biosorption equilibrium between the metal ion solution and the sorbents (solid phase). It is used to obtain the maximum adsorption capacity related to complete monolayer coverage on the homogeneous surface of the sorbent^22^. The Langmuir equation is given by the following:

3 $$ q_{e} = \frac{{q_{max} K_{L} C_{e} }}{{1 + K_{L} C_{e} }} $$

where *q*_*e*_ (mg/g) is the amount of adsorbed metal ions per gram of sorbent at equilibrium, *q*_*max*_ is the maximum adsorption capacity, *K*_*L*_ (L/g) is the Langmuir constant, which refers to the energy of the adsorption/desorption process, and *C*_*e*_ is the equilibrium metal ion concentration (mg/L)^35,36^.

### Calculation details

All the studied structures were subjected to optimization with G09 program at Spectroscopy Department, National Research Centre, Egypt^37^. B3LYP/6-31 g(d,p) model^38–40^ was used in the calculations. The molecular ESP was calculated at the same level of theory as a test for the reactivity of the studied structures.

## Results and discussion

### Characterization of the biosorbent

#### Fourier transform infrared analysis (FTIR)

FTIR analysis was applied to raw and chemically modified ZSCs in order to characterize the chemical functional groups that are responsible for the removal process.

**Figure 1:**
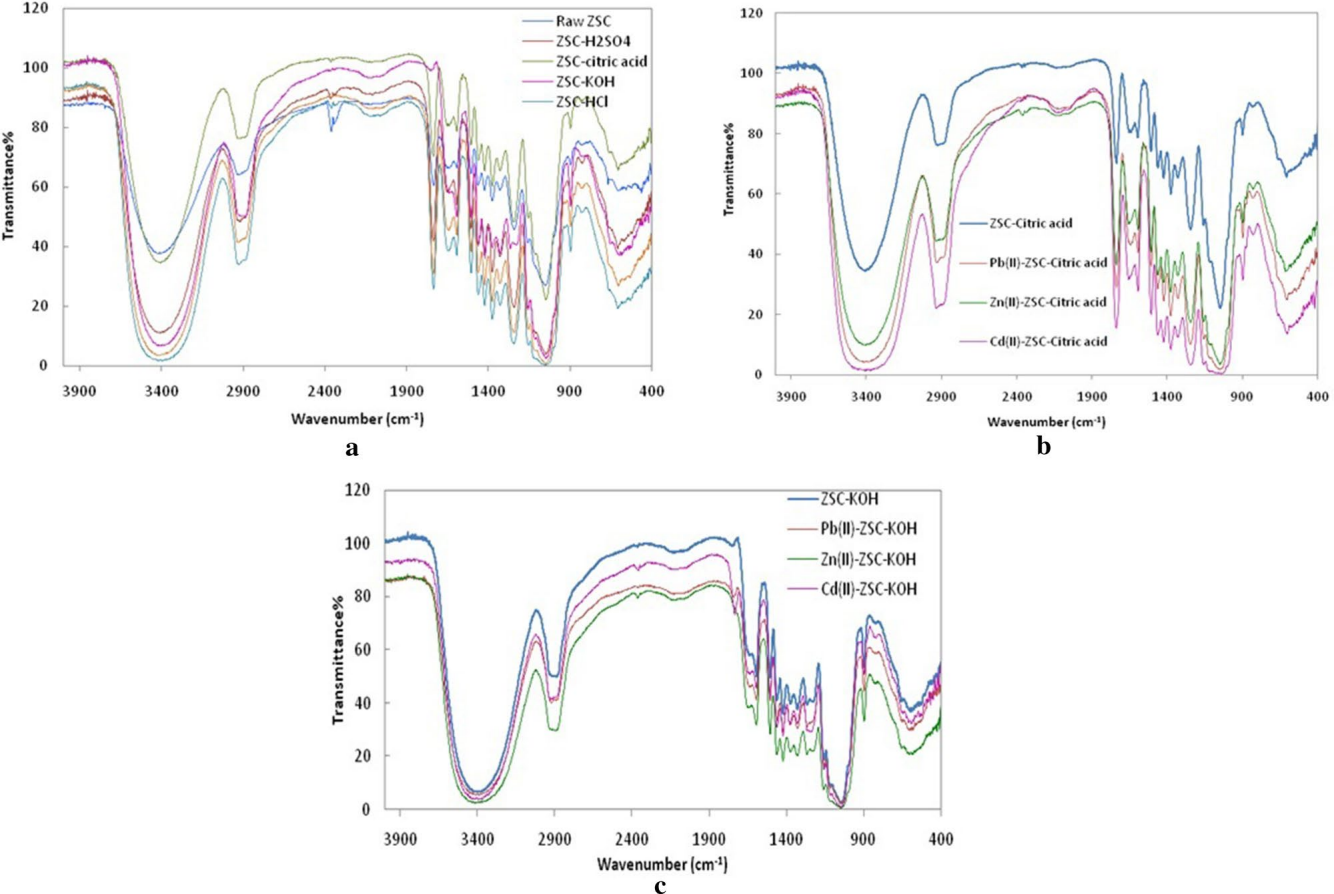
FTIR spectroscopy for (**a**) samples of the raw and chemically modified ZSCs, (**b**) for samples after Pb(II), Zn(II) and Cd(II) ion biosorption by ZSC–citric acid, (**c**) samples after Pb(II), Zn(II) and Cd(II) ion biosorption by ZSC–KOH.

The FTIR spectra of chemically modified ZSCs are shown in Fig. 1. ZSCs modified by diluting acid solutions indicated only a partial modification of their functional group properties. The main bands of raw ZSCs are preserved with slight changes in the intensities of these bands. All acidic-modified ZSCs showed an increase in the broadening of the O–H group at 3356 cm^−1^ with a slight shift indicating the increase in carboxylic O–H groups^41^. An increase in the intensity of the C = O band at 1730 cm^−1^ also indicated the increase in carboxylic groups. In addition, there was some increase in the intensity of the bands at 1035–1134 cm^−1^, demonstrating an increase in the functional groups of C–O stretching vibrations such as ethers and phenols^42^. The FTIR spectra of ZSCs chemically modified by diluting alkali solution showed a shift of the C=O band (1730 cm^−1^) and slight shifting of the band of carboxylate ion (1610 cm^−1^) and the band of the O–H group (3356 cm^−1^) to lower wavenumbers due to the hydrolysis of ester groups, which subsequently increased the carboxylate groups and O–H group^43^. It is also noted that the C–O band intensity (at 1230 cm^−1^) decreased, indicating the removal of waxes by the alkali treatment^44,45^. In the case of citric acid modification, an esterification reaction may have occurred due to the shift of the band at 3356 cm^-1^ to 3340 cm^−1^and the sharpening of the band at 1730 cm^−1^^46,47^. Hydrogen peroxide modification shows an increase in the intensity of the bands at 1730 cm^−1^and 1035 cm^−1^, indicating partial oxidation of C=O and C–O functional groups^4,8^.

Figure 1a–c present the FTIR spectroscopy for raw samples as well as other samples after metals biosorption. The differences observed in the spectra after metal ion biosorption, as shown in Fig. 1 b,c, were around 1035, 1230, 1134 and 1464–1610 cm^−1^, which are related to the changes in the vibrations of C–O and –COO− functional groups. This means that chelating Pb(II), Zn(II) and Cd(II) ions by ZSCs may be attributed to the phenolic groups of the lignin and/or to the carboxyl groups contained in the ZSCs^23^. The changes in the band at 3356 cm^−1^ means that the hydroxyl group is also involved in the metal ion biosorption^49^. For ZSC–KOH, the shift of the C=O of carboxyl groups from 1705 to 1730 cm^−1^ is an indication for the metal coordination by ZSCs which is in a good agreement with the previous findings^50^.

### Biosorption of Pb(II),Zn(II) and Cd(II) by chemically modified ZSCs

#### Effect of contact time for Pb(II), Zn(II) and Cd(II) removal by chemically modified ZSCs

**Figure 2:**
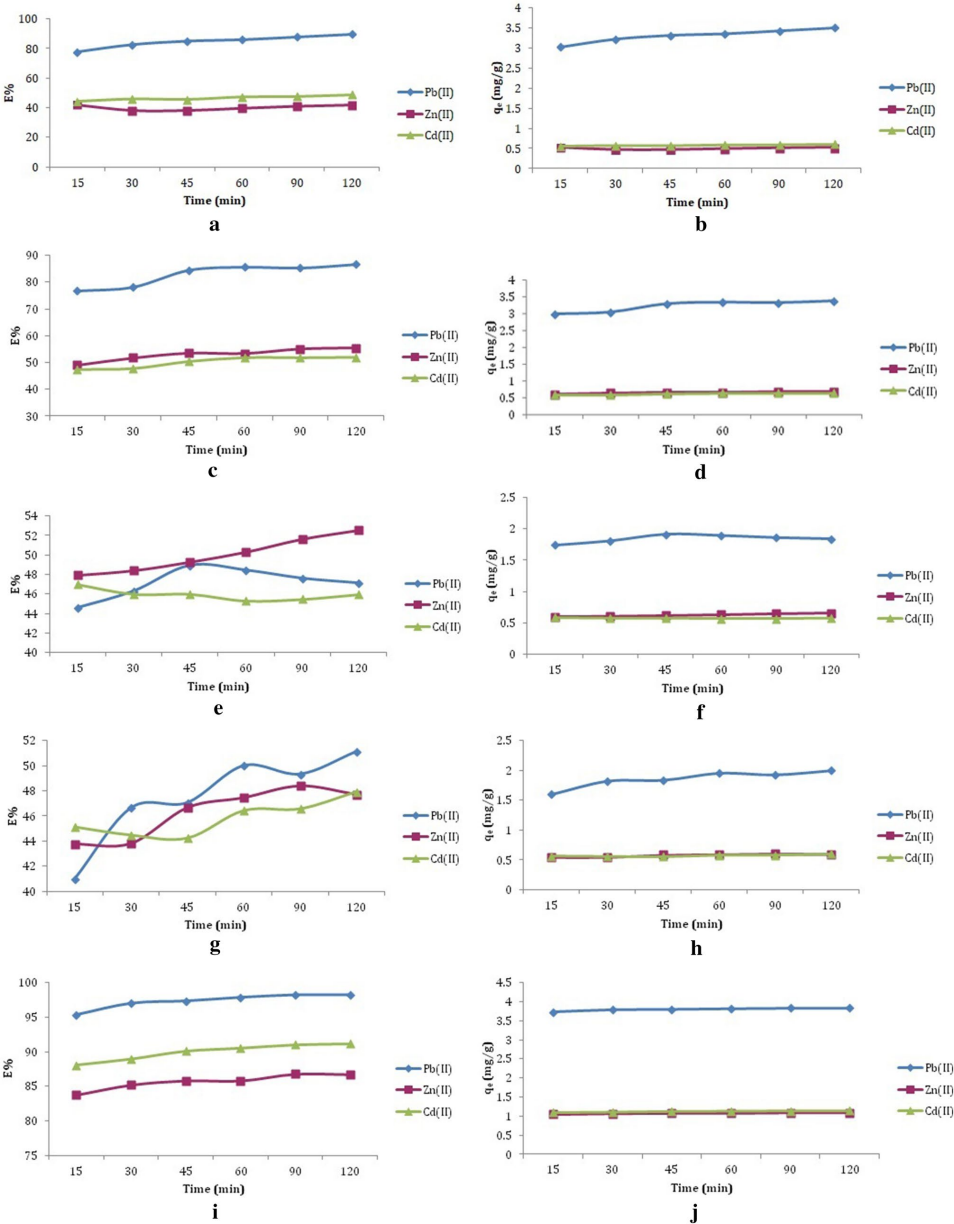
The contact time as a function of the (**a**) removal efficiency (E%) of Pb(II), Zn(II) and Cd(II) ions by ZSC–H_2_SO_4_; (**b**) the amount of biosorbed Pb(II), Zn(II) and Cd(II) ions per gram of ZSC–H_2_SO_4_; (**c**) the removal efficiency (E%) of Pb(II), Zn(II) and Cd(II) ions by ZSC–citric acid; (**d**) the amount of biosorbed Pb(II), Zn(II) and Cd(II) ions per gram of ZSC–citric acid; (**e**) the removal efficiency(E%) of Pb(II), Zn(II) and Cd(II) ions by ZSC–HCl; (**f**) the amount of biosorbed Pb(II), Zn(II) and Cd(II) ions per gram of ZSC–HCl; (**g**) the removal efficiency (E%) of Pb(II), Zn(II) and Cd(II) ions by ZSC–H_2_O_2_; (**h**) the amount of biosorbed Pb(II), Zn(II) and Cd(II) ions per gram of ZSC–H_2_O_2_; (**i**) the removal efficiency(E%) of Pb(II), Zn(II) and Cd(II) ions by ZSC–KOH and (**j**) the amount of biosorbed Pb(II), Zn(II) and Cd(II) ions per gram of ZSC–KOH.

The effect of contact time was studied for the five different chemically modified ZSCs: Modified by H_2_SO_4_, citric acid, HCl, H_2_O_2_ and KOH, at the studied time intervals of 15, 30, 45, 60, 90, 120 min, using 0.2 g of ZSCs with a size of 250 μm, and initial metal ion concentration of 31.27 mg/L for Pb(II) and 10 mg/L for Zn(II) and Cd(II) ion solution at a pH of 6 (Fig. 2a–j).

The removal percentage (%E) of metal ions by modifying ZSCs was rapid initially, then increased slightly with contact time until reaching equilibrium. The equilibrium time was attained at 60 min in all cases. The biosorption capacity also slightly increased with time until it reached equilibrium. There was no difference between raw and modified ZSCs in terms of the effect of time on. A comparison between the removal of Pb(II), Zn(II) and Cd(II) by raw and modified ZSCs at equilibrium is shown in Table 1.

**Table 1:** The biosorption process for the Pb(II), Zn(II) and Cd(II) ions by raw and modified ZSCs at equilibrium time.

Biosorbent	%E		
	Pb(II)	Zn(II)	Cd(II)
Raw ZSC	71.43	41.84	39.25
ZSC–HCl	48.45	50.30	45.37
ZSC–H_2_O_2_	49.98	47.45	46.42
ZSC–H_2_SO_4_	85.96	39.61	47.45
ZSC–citric acid	85.71	53.33	51.80
ZSC–KOH	97.88	85.80	90.56

From the results shown in Table 1, it was observed that the modification enhanced the process of metal removal in all cases except for the removal of Pb(II) ions by HCl and H_2_O_2_-modified ZSCs, and the removal of Zn(II) by H_2_SO_4_-modified ZSCs. These had a negative effect on the efficiency of removal. The enhancement of Pb(II) removal followed the order ZSC–KOH > ZSC–H_2_SO_4_ < ZSC–citric acid. Zn(II) ion removal followed the order ZSC–KOH > ZSC–citric acid > ZSC–HCL < ZSC–H_2_O_2_. Finally, the Cd(II) removal trend was ZSC–KOH < ZSC–citric acid < ZSC–H_2_SO_4_ < ZSC–H_2_O_2_ < ZSC–HCl. The modified ZSCs by KOH gave the most remarkable enhancement of removal capacity in all cases. This may be because alkali modification can increase the biosorption capacity by removing the materials that block the binding sites, such as protein and lipids^51^. Potassium hydroxide is a good reagent for the conversion of the ester groups to carboxylate and alcohol groups, which is favorable for cationic metal attraction^52,53^. Acidic modification also can increase the biosorbent surface area and porosity by eliminating the impurities, leading to an enhanced biosorption capacity^54,55^. Acidic modification also induces hydrolysis of the biosorbent functional groups and raises the anionic binding sites for cationic metals^51^.

#### Biosorption isotherm for Pb(II), Zn(II) and Cd(II) by chemically modified ZSCs

The Langmuir isotherm model was applied to calculate the maximum adsorption capacity related to the complete monolayer formation on the surface of modified ZSCs. The data were obtained using an initial metal ion concentration range of 2.5–31.27 mg/L, with a solution with a pH of 6 and 0.2 g of chemically modified ZSCs with a size of 250 μm that was shaken for 60 min.

**Figure 3:**
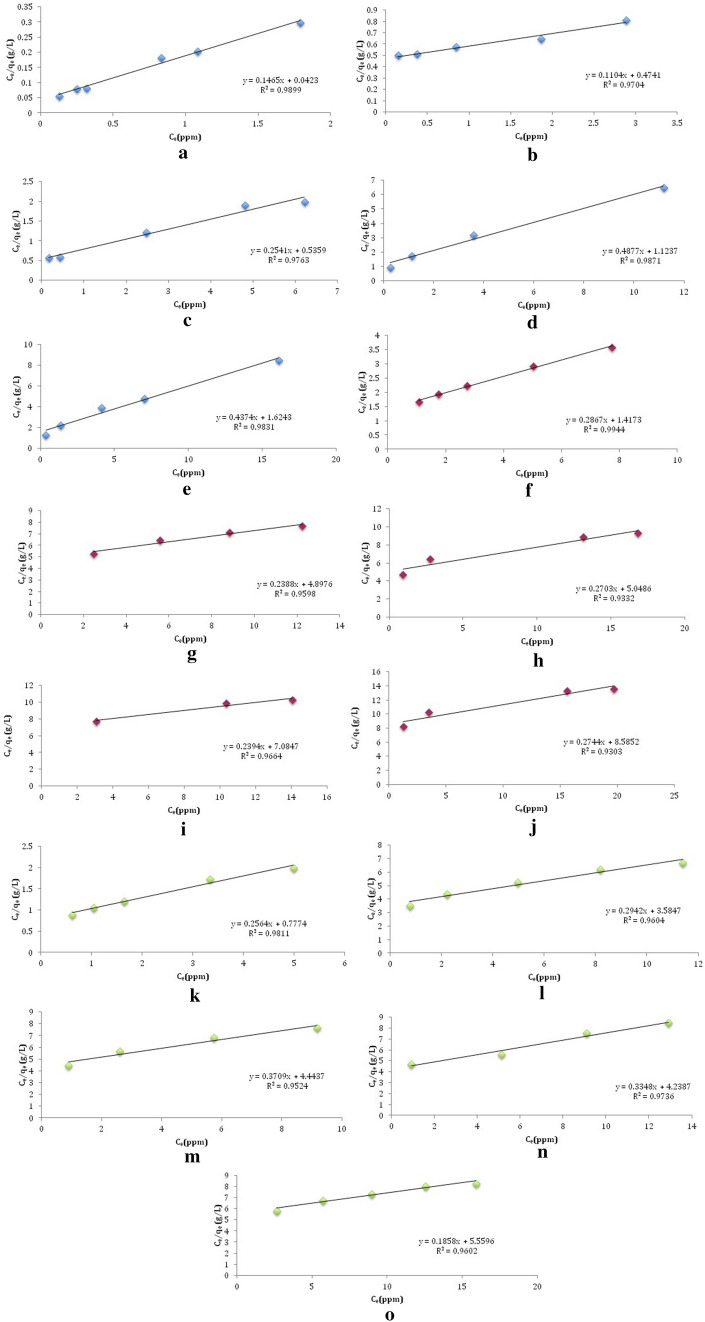
Langmuir isotherm for the biosorption of the following (**a**) Pb(II) onto ZSC–KOH; (**b**) Pb(II) onto ZSC–citric acid; (**c**) Pb(II) on to ZSC–H_2_SO_4_; (**d**) Pb(II) onto ZSC–HCl; (**e**) Pb(II) onto ZSC–H_2_O_2_; (**f**) Zn(II) onto ZSC–KOH; (**g**) Zn(II) onto ZSC–citric acid; (**h**) Zn(II) onto ZSC–H_2_SO_4_; (**i**) Zn(II) onto ZSC–HCl; (**j**) Zn(II) onto ZSC–H_2_O_2_; (**k**) Cd(II) onto ZSC–KOH; (**l**) Cd(II) onto ZSC–citric acid, (**m**) Cd(II) onto ZSC–H_2_SO_4_; (**n**) Cd(II) onto ZSC–HCl and (**o**) Cd(II) onto ZSC–H_2_O_2_.

Linearity plots, as presented in Fig. 3a–o, illustrate that the Langmuir model was acceptable to represent the biosorption isotherm. Table 2 shows that the highest value of the maximum adsorption capacity on the modified ZSCs for Pb(II) was 9.06 mg/g and 4.19 mg/g for Zn(II) obtained by using ZSC–citric acid, while for Cd(II), it was 5.38 mg/g obtained by using ZSC–H_2_O_2_.

**Table 2:** Langmuir isotherm parameters for the biosorption of Pb(II), Zn(II) and Cd(II) onto the modified ZSCs.

Metals ion, ZSC type	Langmuir isotherm parameters			
	*q*_*max*_ (mg/g)	*K*_*L*_(L/g)	*R*^2^	*R*_*L*_
Pb–ZSC–KOH	6.83	3.46	0.9899	0.01
Pb– ZSC–citric acid	9.06	0.23	0.9704	0.12
Pb– ZSC–H_2_SO_4_	3.94	0.47	0.9762	0.06
Pb– ZSC–HCl	2.05	0.43	0.9871	0.07
Pb– ZSC–H_2_O_2_	2.29	0.27	0.9830	0.11
Zn– ZSC–KOH	3.49	0.20	0.9944	0.14
Zn– ZSC–citric acid	4.19	0.05	0.9597	0.40
Zn– ZSC–H_2_SO_4_	3.70	0.05	0.9332	0.37
Zn– ZSC–HCl	4.18	0.03	0.9664	0.49
Zn– ZSC–H_2_O_2_	3.64	0.03	0.9303	0.50
Cd– ZSC–KOH	3.90	0.33	0.9811	0.09
Cd– ZSC–citric acid	3.40	0.08	0.9604	0.28
Cd– ZSC–H_2_SO_4_	2.70	0.08	0.9523	0.28
Cd– ZSC–HCl	2.99	0.08	0.9736	0.29
Cd– ZSC–H_2_O_2_	5.38	0.03	0.9601	0.49

The Langmuir isotherm characteristics can be described by the equilibrium parameter (R_L_)^4,6^:

4 $$ R_{L } = \frac{1}{{\left( {1 + K_{L} C_{0} } \right)}} $$

where *K*_*L*_ is the Langmuir constant and *C*_0_ is the initial sorbate concentration (mg/L). An *R*_*L*_ value that is > 0 and < 1 shows a favorable isotherm, if *R*_*L*_ = 1, the isotherm would be linear, and if *R*_*L*_ > 1 the isotherm would be unfavorable^56^. The *R*_*L*_ values indicate that biosorption is favorable (0 < *R*_*L*_ < 1).

Lead exhibits a high amount of biosorption and removal efficiency by both raw and chemically modified ZSCs. In fact, Pb(II) and Zn(II) were classified as borderline Lewis acids, and Cd(II) was classified as a soft acid according to hard–soft–acid–base theory. This theory explains the interaction between metal ions and ligands, as the hard acid tends to interact with a hard base, and soft acid with a soft base. Therefore, the increased binding affinity of Pb(II) and, in some cases, Zn(II) in comparison to Cd(II) ions can be attributed to their tendency to bind with the hard carboxylate and hydroxyl base^57^. Heavy metal selectivity also referred to some of their metallic properties^58^. Lead has a larger ionic radius size (1.33 Å) compared with Zn(II) (0.74 Å) and Cd(II) (0.97 Å), which means that Pb(II) ions are less hydrated than Zn(II) and Cd(II)^59,60^. It has also higher electronegativity, resulting in a harder Lewis acid than zinc and cadmium^61^. From the previously determined Langmuir constant K_L_, it is observed that Pb(II) ions have more affinity to the raw and chemically modified ZSCs than Zn(II) and Cd(II) ions.

#### Mechanism of biosorption of Pb(II), Zn(II) and Cd(II) by chemically modified ZSCs

In order to understand the mechanism of interaction between ZSCs and the studied metals, density-functional theory (DFT) was conducted at DFT: B3LYP level with 6-31 g(d,p) basis set.

**Figure 4:**
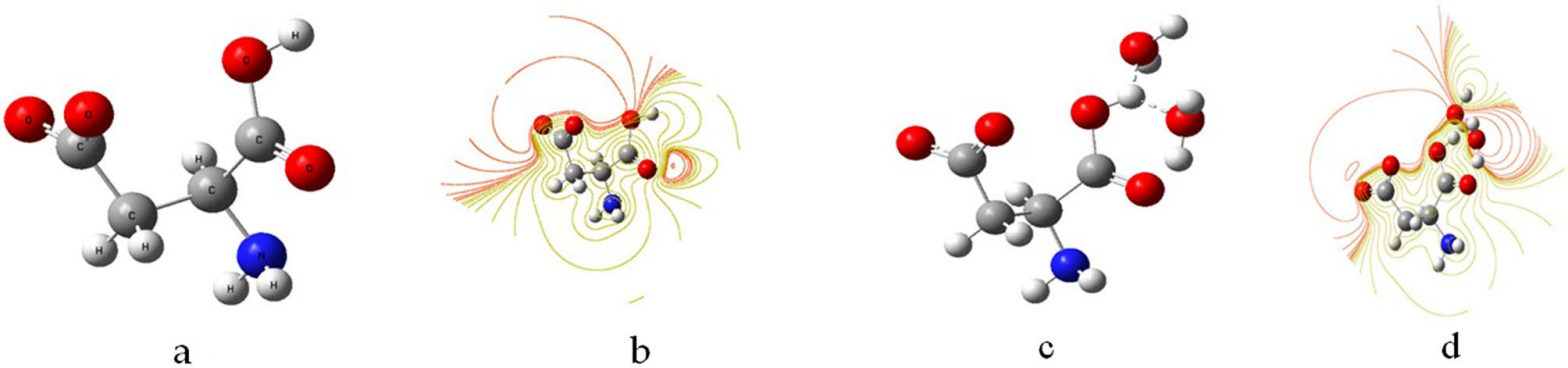
Model molecules for (**a**) aspartic acid as a model molecule for ZSCs, (**b**) ESP contour for aspartic acid, (**c**) di-hydrated aspartic acid, (**d**) ESP contour for di-hydrated aspartic acid.

Aspartic acid was proposed to simulate the ZSCs molecules. Therefore, the coordination of the metal with ZSCs could be the result of coordination with COOH, which is typical for organic acids as well as amino acids with a protein structure. In this part, the coordination of Cd and Zn is attempted. The model molecules shown in Fig. 4 show that aspartic acid and its molecular ESP as a contour are described; then, the di-hydration of the acid is also described after the interaction with two water molecules, and subsequently the contour of ESP. The dependence on the ESP as a marker for reactivity was described earlier^62–65^. The red color is an indication of active sites, such that the active sites in Fig. 4b–d is more likely to occur around the carboxyl group as compared to the amide group.

**Figure 5:**
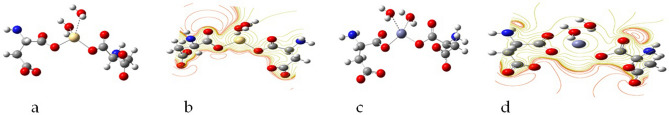
Model molecules for (**a**) di-hydrated cadmium interacting with two aspartic units, (**b**) ESP contour for di-hydrated cadmium interacting with two aspartic units, (**c**) di-hydrated zinc interacting with two aspartic units, (**d**) ESP contour for di-hydrated zinc interacting with two aspartic units.

Figure 5 demonstrates the model molecules for a) di-hydrated cadmium interacting with two aspartic units, and b) the ESP contour for di-hydrated cadmium interacting with two aspartic units. It is clear that Cd ions interacted weakly with two water molecules, then further interacted with two carboxyl groups of two aspartic units as a complex interaction. The contour of ESP shows an increase in the active site around the carboxyl with less activity around the amide. Another confirmation of the mechanism is conducted in the case of di-hydrated Zn, as shown in Fig. 5, which shows the model molecules for c) di-hydrated zinc interacting with two aspartic units, and d) the ESP contour for di-hydrated zinc interacting with two aspartic units.

Zinc was also di-hydrated, then interacted with the carboxyl of two aspartic units through the hydrogen bonding as a complex interaction.

The ESP is also more likely to occur around the carboxyl group. As compared to the results of FTIR, the molecular modeling work suggested that the probability of the mediation of heavy metals with ZSCs is more likely to happen through the COOH of the amino acids.

The presented model describes the mechanism of interaction between the plant and the hydrated metals. Other efforts which describe the biosorption with Ziziphus could be found recently in the literature^66–68^. This work gave insight into another possibility for the plant to remediate other pollutants rather than HMs.

## Conclusions

Green methods for controlling water pollution is very important as it is depending on natural resources. This route is minimizing the adverse impacts on the aquatic environment. For this purpose, the natural materials ZSCs were chemically modified by testing different alkaline and acidic chemicals. FTIR analysis indicated that the raw lignocellulosic ZSCs consisted of different oxygen-containing functional groups, allowing it to possibly link to the metal ions either by ion-exchanging or complexation. Alkali-modified ZSCs by KOH in comparison with the acidic ones was more effective and enhanced the ZSCs removal efficiency by increasing the carboxylate ions. The biosorption of Pb(II), Zn(II) and Cd(II) ions on the chemically modified ZSCs followed the Langmuir isotherm model. The highest value of the maximum adsorption capacity of the modified ZSCs for Pb(II) was 9.06 mg/g, and 4.19 mg/g for Zn(II) as obtained by using ZSC–citric acid, while for Cd(II), the highest value was 5.38 mg/g, obtained by using ZSC–H_2_O_2_. The chemically modified ZSCs can be used as an efficient biosorbent for heavy metals remediation from wastewater, as an eco-friendly and economic material which is more selective for Pb(II) removal. The molecular modeling study indicated that each metal is di-hydrated, then formed a stable complex with two amino acid units. Therefore, the mechanism of uptake is described as a complex state between the protein structure of the ZSCs and the hydrated heavy metals.
